# Unimodal and multimodal regions for logographic language processing in left ventral occipitotemporal cortex

**DOI:** 10.3389/fnhum.2013.00619

**Published:** 2013-09-27

**Authors:** Yuan Deng, Qiuyan Wu, Xuchu Weng

**Affiliations:** ^1^Key Laboratory of Behavioral Science, Institute of Psychology, Chinese Academy of SciencesBeijing, China; ^2^Center for Cognition and Brain Disorders, Hangzhou Normal UniversityHangzhou, China

**Keywords:** fMRI, visual word form area, Chinese, multimodal, task modulation

## Abstract

The human neocortex appears to contain a dedicated visual word form area (VWFA) and an adjacent multimodal (visual/auditory) area. However, these conclusions are based on functional magnetic resonance imaging (fMRI) of alphabetic language processing, languages that have clear grapheme-to-phoneme correspondence (GPC) rules that make it difficult to disassociate visual-specific processing from form-to-sound mapping. In contrast, the Chinese language has no clear GPC rules. Therefore, the current study examined whether native Chinese readers also have the same VWFA and multimodal area. Two cross-modal tasks, phonological retrieval of visual words and orthographic retrieval of auditory words, were adopted. Different task requirements were also applied to explore how different levels of cognitive processing modulate activation of putative VWFA-like and multimodal-like regions. Results showed that the left occipitotemporal sulcus (LOTS) responded exclusively to visual inputs and an adjacent region, the left inferior temporal gyrus (LITG), showed comparable activation for both visual and auditory inputs. Surprisingly, processing levels did not significantly alter activation of these two regions. These findings indicated that there are both unimodal and multimodal word areas for non-alphabetic language reading, and that activity in these two word-specific regions are independent of task demands at the linguistic level.

## Introduction

Extensive evidence from imaging studies has shown that a region in the human left extrastriate visual cortex responds selectivity to written letters over other complex visual stimuli, such as line-drawings, faces, and houses, and that these responses are highly invariant with changes in visual script or font (Cohen et al., 2000, 2002, Cohen and Dehaene, 2004; Dehaene et al., 2001, 2002, 2005, 2010; Szwed et al., 2011). This region, located lateral to the middle part of the left fusiform gyrus, was labeled the visual word form area (VWFA; Cohen et al., 2000; Dehaene and Cohen, 2011). However, controversies remain about this region's function in reading and reading development. The main point of debate is whether the specialization of the VWFA is domain specific (Dehaene and Cohen, 2011) or process-specific (Price and Devlin, 2011).

In contrast to the view of visual-specific selectivity, the interactive view suggests that this region may act as an interface between sensory input and higher level associations (e.g., mapping visual word forms to sounds and meanings) (Price and Devlin, 2011), as functional connectivity studies have shown that the left fusiform gyrus interacts extensively with other regions of the reading network. When there was a strong demand for linguistic judgment, activation of this region was highly correlated with activation of regions associated with semantic and phonological processing (Bitan et al., 2005; Wang et al., 2011) as well as visuospatial processing of logographic writing systems (Deng et al., 2012). Evidence from lexical training studies has shown that the left mid fusiform region is critical for new script learning (Hashimoto and Sakai, 2004; Deng et al., 2008; Dehaene et al., 2010) and that activation of this region increases during phonological and semantic learning of a new script (Sandak et al., 2004; Xue et al., 2006). A recent publication found that congenitally blind subjects exhibited VWFA activation when selectively doing a letter-soundscapes task, suggesting that the VWFA may be responsible for linking letter shape to phonology (Striem-Amit et al., 2012).

Cohen et al. (2004) further verified the exclusive response of the VWFA to visual inputs by directly examining the modality effect in the left temporo-occipital region, and proposed that an adjacent region, the lateral inferotemporal multimodal area (LIMA), showed comparable activation for both visual and auditory inputs. A similar pattern of activation was found by Jobard et al., (2003, 2007), who labeled this multimodal area the basal temporal language area (BTLA). However, because the alphabetic writing systems used in these studies (English and French) have grapheme-to-phoneme correspondence (GPC) rules, it is difficult to disassociate visual-specific processing from form-to-sound mapping in VWFA activation for visual word recognition. These GPC rules may also contribute to the distinct spatial organization of unimodal and multimodal regions in the left inferotemporal cortex of alphabetic language speakers.

Compared to alphabetic language systems, a typical logographic language (such as Chinese) does not follow GPC rules for word form-to-sound mapping. Chinese characters map onto phonology at the mono-syllable level, and the relationship is usually arbitrary. For example, the character 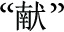 (“contribute”) is pronounced /xian4/ (the number refers to tone), thus no visual component of this character corresponds to a phoneme of character pronunciation. This lack of systematic mapping between visual form and phonology makes Chinese script a unique tool to control for the possible confound of sub-lexical form-to-sound processing by the VWFA and (or) associated cortical regions.

Thus, by taking advantage of this unique characteristic of the Chinese language, the current study aimed to examine the following issues regarding the function of the VWFA. First, without simultaneously changing phonological affordances of the stimuli, can different levels of phonological processing of Chinese characters influence VWFA activation? Second, is the VWFA a unimodal region in logographic word reading as it is in languages with GPC rules? Is there also a multimodal region in the ventral temporal cortex for Chinese character reading? Is the VWFA activated during auditory word processing when requiring access to orthographic representations in a logographic language system? Finally, can different orthographic retrieval requirements influence activation of VWFA and/or the putative multimodal region?

In order to answer these questions, the current study employed both visual-to-auditory and auditory-to-visual cross-modality tasks, and modulated task demands for phonological retrieval and character-form retrieval at different sub-lexical levels. If activation of the VWFA is modulated by phonological retrieval at different sub-lexical levels for Chinese reading, it suggests that attention to sub-lexical processing can indeed confound the response properties of the VWFA regardless of the form-to-sound mapping principle. The opposite result suggests that form-to-sound mapping in the VWFA happens at the mono-syllable level, at least for reading Chinese characters. Moreover, if there is a distinct sub-region in the left inferotemporal region that shows comparable activation for both auditory and visual inputs, it may suggest a common multimodal region across writing systems. To our knowledge, this is the first study to directly examine the universality of unimodal/multimodal regions in the ventral temporal cortex.

## Materials and methods

### Subjects

Fifteen native Chinese speakers (19–25 years old) participated in this study. All participants were undergraduate or graduate students. Participants were right-handed and had normal hearing and normal or corrected-to-normal vision. They gave informed consent in accordance with guidelines set by the Beijing MRI Center for Brain Research, China.

### Tasks and materials

#### Experimental design

**Figure 1:**
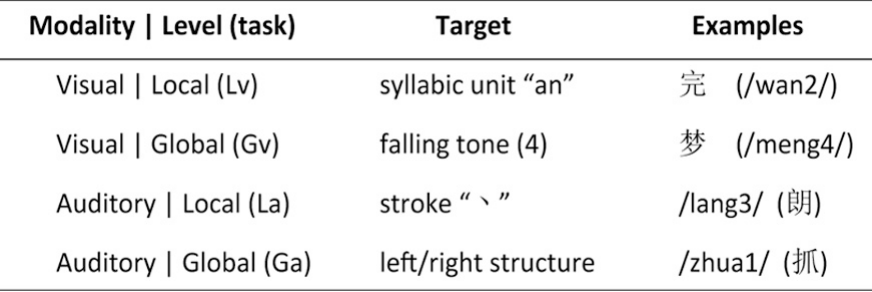
**Experimental design**. Chinese pronunciations (pinyin) for visual-based tasks and Chinese characters for auditory-based tasks are displayed in parenthesis.

As shown in Figure 1, a 2 input-modality (visual and auditory) × 2 processing-level (local and global) within-subject design was adopted. There were four experimental tasks: syllabic-unit judgment (local-level) for visual words (Lv), tone judgment (global-level) for visual words (Gv), stroke judgment (local-level) for auditory words (La), and structure judgment (global-level) for auditory words (Ga).

Furthermore, a perceptual task was used in an independent scanning session (localizer task) in order to localize the word-specific region for Chinese scripts in the occipitotemporal area (Ma et al., 2011).

#### Stimuli in visual-based tasks

One hundred and sixty single-character Chinese words were selected from a pool of the most commonly used characters according to the Modern Chinese Frequency Dictionary (see Supplementary Material). Half of them (80) were used for each task. There was only one phonological correspondence for each visual character, i.e., these characters were not polyphones. The average stroke number of these characters was 9.55 (*SD* = 2.40), suggesting a medium visual complexity. For both Lv and Gv tasks, all characters were presented in black against a white background in Song font (100 × 100 pixels).

In the Lv task, participants determined whether the pronunciation of a character contains the syllabic unit “an.” In the Gv task, participants determined whether the character has a falling tone (the fourth tone in Chinese). For both tasks, participants made the yes/no decision by pressing the right or left button on a response box. A perceptual task served as a control. In this task, participants determined whether a caret-like character (/\) was present on the left of a line drawing (/\ \) or on the right (//\). They made the left/right decision by pressing the left or right button. There were a total of one hundred and sixty line drawings, eighty for the Lv task and eighty for the Gv task.

#### Stimuli in auditory-based tasks

The Chinese language consists of a very large number of homophones, sounds represented by several different (visual) word forms. A key consideration in selecting stimuli for tasks of auditory-based word-form judgment was to make sure that only one specific visual correspondence (character) could be retrieved for each auditory word. To this end, a group of characters that have no homophones or few (low frequent) homophones were chosen. Then, another 30 subjects from the same sample group, who did not participate the functional magnetic resonance imaging (fMRI) experiment, were asked to write down the character(s) that first came to their mind when they listened to a speech sound. Only those speech sounds that showed high consistence and accuracy (recognizability) were chosen as final stimuli. Due to these limitations, a total of 80 Chinese single-character words were selected for both tasks, i.e., the La and Ga task used the same set of stimuli (see Supplementary Material). According to the dictionary, the majority of final stimuli had no homophones, while some characters (24 out of 80) had a few homophones with extremely low frequency. All stimuli were presented in an auditory format. All auditory words were recorded in a soundproof booth using a digital recorder and a high-quality stereo microphone. A native Chinese woman read aloud each pronunciation in isolation. Sound duration was normalized to 800 ms and presented at the same sound intensity (loudness).

In the La task, participants determined whether the written form of an auditory word contains a specific 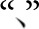 (dot) stroke. In the Ga task, participants determined whether the written structure of an auditory word has a left-right structure, i.e., whether two major visual components of a character are horizontally configured. Again, they made the yes/no decision by pressing the right or left button on the response box. A perceptual task served as a control. In this task, participants were asked to judge whether the volume of the tone was low, and made the yes/no decision by pressing the right or left button.

#### Validation of experimental tasks

In order to test the validity of these tasks (i.e., different requirements for sub-lexical processing induce different psycholinguistic processing levels), a pilot behavioral study was conducted. Ten subjects from the same sample group, who did not participate in the fMRI experiment, were asked to complete all four tasks. Result showed that subjects performed significantly faster in the global condition (mean RT: 1477.9 ms) than in the local condition (mean RT: 1887.2 ms) in auditory-based tasks [*t*_(9)_ = −10.938, *p* < 0.001]. For visual-based tasks, subjects also demonstrated a consistent trend for better performance in the global condition (mean RT: 1240.6 ms) compared to the local condition (mean RT: 1363.9 ms). Faster performance in the global condition is in accordance with the classic finding of “global precedence” in the domains of visual perception (Navon, 1977), attention (Miller, 1981), and mental imagery (Qiu et al., 2009; Niu and Qiu, 2013), indicating that the tasks employed do indeed require different levels of cognitive processing. In addition, although the global-local difference in visual tasks was not as large as in the auditory tasks, evidence from brain imaging studies have consistently found that phoneme/syllabic-unit processing activated a different neural network compared to supersegmental processing (e.g., tones) in Chinese (Gandour et al., 2003; Tong et al., 2008; Li et al., 2010). Evidence from a brain connectivity study also found that distinct brain networks were engaged by global and local information processing for mental imagery (a paradigm similar as our auditory-based task) (Li et al., 2008).

#### VWFA localizer

The stimuli and procedures were adapted from a previous study (Ma et al., 2011). Three categories of stimuli, including Chinese characters, faces, and line-drawings, were used. The stimuli were chosen randomly from a pool of 80 during the experiment. Within each trial, the center of each stimulus was slightly shifted from the center of the fixation point and participants were asked to make a judgment about whether the center of the picture was to the left or the right compared to the fixation point by pressing the left/right button.

### fMRI procedures and timing

All participants practiced a short version of each experimental task before the fMRI scanning session. Different stimuli were used in the practice and the fMRI sessions. There were a total of six functional scanning runs for each subject, including four runs for experimental tasks (Lv, Gv, La, Ga) and two runs for localizer tasks.

For all four experimental runs, a block design was used for stimulus presentation. There was one run for each task. The task order was counterbalanced across subjects. Each run consisted of four experimental task blocks and 4 control task blocks. Each trial lasted 2 s. There were 20 trials per block, and a 2 s instruction trial before each block, so each experimental run lasted 336 s.

After four experimental task runs, there were two identical localizer runs. Each localizer run consisted of 3 blocks repeated three times, one block for each of the three stimulus categories (characters/faces/line-drawings). The block order for the three categories was pseudo-randomized, with a 20 s fixation interval between successive blocks. Each block involved the presentation of 20 images (each for 250 ms), interleaved with a central fixation cross shown for 750 ms. Therefore, each localizer run lasted 380 s.

### Image acquisition

Brain images were obtained on a 3T Siemens Trio scanner at the Beijing MRI Center for Brain Research. Participants lay in the scanner with their head position secured with a specially designed vacuum pillow. Participants were asked to hold an optical response box. The head coil was positioned over the participants' head. Participants viewed visual stimuli that were projected onto a screen via a mirror attached to the inside of the head coil and listened to auditory stimuli via earphones.

For the functional imaging runs, a susceptibility weighted single-shot echo planar imaging (EPI) method with blood oxygenation level-dependent (BOLD) was used. The following scan parameters were used: *TE* = 35 ms, flip angle = 90°, matrix size = 64 × 64, field of view = 24 cm, slice thickness = 4 mm, number of slices = 32, *TR* = 2000 ms. In addition, a high resolution, T1-weighted 3D image was acquired (3D MPRAGE; 1.33 × 1 × 1 mm^3^ resolution, 144 slices and 1.33 mm slice thickness with no gap).

### Data analysis

Data analysis was performed using BrainVoyager QX 2.0 software (Brain Innovation; Goebel et al., 2006). Due to technical problems, data from four subjects were excluded from the final analysis. The functional images were preprocessed; preprocessing steps included slice scan timing correction, motion correction with respect to the first volume in the run, and high-pass filtering (2 cycles per series cutoff). Functional data were not smoothed. Preprocessed functional data were then coregistered to high-resolution anatomical images, which in turn were normalized to Talairach space (Talairach, 1988). Normalizations were performed by using a piecewise affine transformation based on manual identification of the anterior and posterior commissures and the edges of cortex along each axis on anatomical data.

Data from all four experimental runs for each participant were entered into a general linear model using a block analysis procedure. Parameter estimates from BOLD contrasts in single participant model were entered into a random-effects model for all participants to determine whether activation was significant for a contrast at the group level. To reveal overall activation patterns for visual and for auditory stimuli, two tasks of the same modality were combined (Lv and Gv for visual, La and Ga for auditory). The threshold was set at *p* < 0.05 FDR-corrected with a cluster size of 10 voxels or greater. Differences between each condition were also examined by paired *t*-test. Statistical threshold was set at *p* < 0.001 and cluster-size threshold estimation was performed for correction of multiple comparisons.

Based on two localizer runs, regions-of-interest (ROIs) in the ventral visual pathway for visual word-form processing were selected. According to a previous study (Ma et al., 2011), the contrast between Chinese characters and faces was used to localize the region showing higher activation for words (FDR-corrected, *p* < 0.05). At the single subject level, two regions in the left ventral temporal region showed significantly greater activation for Chinese characters, and this activation pattern was consistent across subjects. Based on the anatomical location of these activated regions, the following two ROIs were recognized: the left occipitotemporal sulcus (LOTS) and the left inferior temporal gyrus (LITG). Accordingly, each participant's individual ROIs were identified with the exception of one participant who showed a similar cortical activation pattern in response to Chinese characters and faces. The mean estimates of ROI activation (Beta value) for each subject and for each experimental task (Lv, Gv, La, Ga) relative to control tasks were then obtained using the ROI GLM tool in the BVQX package. Finally, these data were entered into a 2 region (LOTS and LITG) × 2 input-modality (visual and auditory) × 2 processing-level (local and global) ANOVA analysis.

## Results

### Behavioral results

Due to technical problems, the data from four of the 15 participants were not included in the final analysis. The average accuracies were 95.6% for Lv, 91.5% for Gv, 79.1% for La, and 87.5% for Ga. The reaction times (RTs) were 658 ms for Lv, 696 ms for Gv, 885 ms for La, and 902 ms for Ga. Significant main effects of input modality were found for both accuracy [*F*_(1, 10)_ = 41.827, *p* < 0.001] and RT [*F*_(1, 10)_ = 35.305, *p* < 0.001], suggesting that participants performed better and responded faster in visual tasks than auditory tasks. A two-way interaction was found for accuracy [*F*_(1, 10)_ = 74.391, *p* < 0.001]. *Post-hoc* analysis showed that participants responded more accurately on global judgments than local judgments for auditory tasks [*t*_(10)_ = 7.149, *p* < 0.001], while there was no significant performance difference between global and local processing for visual tasks. These findings are consistent with results from the pilot behavioral study.

### Imaging results

**Figure 2:**
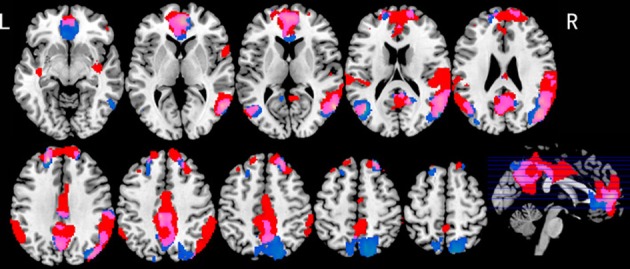
**Brain activation maps for both visual and auditory tasks**. Blue indicates activations for visual tasks; Red indicates activations for auditory tasks; Purple indicates overlapping activations for both tasks. *p* < 0.05, FDR corrected, greater than 10 voxels.

Table 1 shows those areas significantly activated by each modality-specific task (visual and auditory) relative to the corresponding control task. Table 2 shows direct comparisons of the cortical activation patterns between visual and auditory tasks. Figure 2 presents areas of overlapping activation for both task modalities. As seen in Figure 2, both tasks evoked similar activation patterns in the bilateral superior frontal gyrus, bilateral angular gyrus, and posterior cingulate gyrus. However, phonological judgment of visual inputs (Lv and Gv tasks) significantly activated the bilateral superior parietal region, while orthographic judgment of auditory inputs (La and Ga tasks) significantly activated the bilateral temporoparietal junction, including the right superior temporal gyrus and left supermaginal gyrus.

**Table 1:** **Brain regions showing significant activation for visual and auditory tasks compared to control tasks**.

**Activated regions**	***BA***	**Voxels**	***Z*-value**	***x, y, z* {mm}**
**Lv + Gv > CONTROL**				
**R middle temporal gyrus**/L cingulate gyrus	22/39/31/24	6427	5.15	57, −59, 10/−5, −23, −36
**L superior frontal gyrus**/R medial frontal gyrus	9/10	2650	4.87	−17, 44, 29/1, 52, 2
R superior frontal gyrus	8/9	367	4.72	23, 29, 45
**L middle temporal gyrus**/L superior occipital gyrus	39/19	917	4.53	−41, −66, 23
R superior temporal gyrus	38	43	3.91	37, 4, −22
**R inferior parietal gyrus**/R postcentral gyrus	40/1/2	222	3.47	65, −67, 29
R superior frontal gyrus	10/9	146	3.43	17, 59, 15
L posterior cingulate	23	46	3.39	−9, −47, 13
**La + Ga > CONTROL**				
**R superior frontal gyrus**/L medial frontal gyrus	9/10	4790	5.42	9, 59, 22/−9, 52, 6
**R postcentral gyrus**/R middle temporal gyrus/R supramarginal gyrus	40/21	5272	5.1	61, −24, 19
**R precuneus**/L cingulate gyrus	7/31	4929	4.93	7, −48, 42/−10, −30, 42
R parahippocampal gyrus	34	312	4.48	35, −2, −15
**L inferior parietal lobule**/L superior temporal gyrus/L middle temporal gyrus	40/39/19	1472	4.27	−63, −26, 61
L uvala	–	47	4.08	−27, −82, −22
L insula	13	69	3.95	−39, −15, −5
R superior temporal gyrus	22	100	3.76	57, 9, 1
L middle frontal gyrus	10	19	3.41	−37, 41, 18
L middle frontal gyrus	8	58	3.36	−33, 33, 46
L parahippocampal gyrus	34	13	3.35	−27, 3, −16
R middle frontal gyrus	47	39	3.13	47, 38, −5
R middle temporal gyrus	21	24	3.12	65, −35, −8
R superior occipital gyrus	19	10	2.97	35, −88, 22
R fusiform gyrus	20	11	2.82	61, −12, −22

L, left hemisphere; R, right hemisphere; BA, Brodmann's areas. The first area for multiple regions indicate peaks of activation in the clusters. Talairach coordinates; Significance at p < 0.05, FDR corrected (cluster size>10).

**Table 2:** **Brain regions showing significant differences in activation between visual and auditory tasks**.

**Activated regions**	***BA***	**Voxels**	***Z*-value**	***x, y, z* {mm}**
**Lv + Gv > La + Ga**				
**R superior parietal lobule**/R precuneus	7/19	281	3.95	19, −67, 54
R middle temporal gyrus	37	11	3.57	51, −58, 0
R middle temporal gyrus	39	27	3.39	33, −70, 29
**La + Ga > Lv + Gv**				
**R transverse temporal gyrus**/R superior temporal gyrus/R postcentral gyrus	41/22/43	1188	4.88	51, −19, −9
R superior temporal gyrus	22	33	4.84	59, 9, 1
**R cingulate gyrus**/R precuneus	32/7/31	522	4.73	1, 1, 38
**L inferior parietal lobule**/L supramarginal gyrus	40	116	4.32	−63, −37, 33
R parahippocampal gyrus	34	132	3.59	37, −4, −13
L insula	–	17	3.52	−37, −17, −4
**L postcentral gyrus**/L inferior parietal lobule	40	106	3.48	−59, −22, 21
L precuneus	7	127	3.4	−9, −32, 44

L, left hemisphere; R, right hemisphere; BA, Brodmann's areas. The first area for multiple regions indicate peaks of activation in the clusters. Talairach coordinates; Significance at p < 0.001 corrected at cluster level.

**Figure 3:**
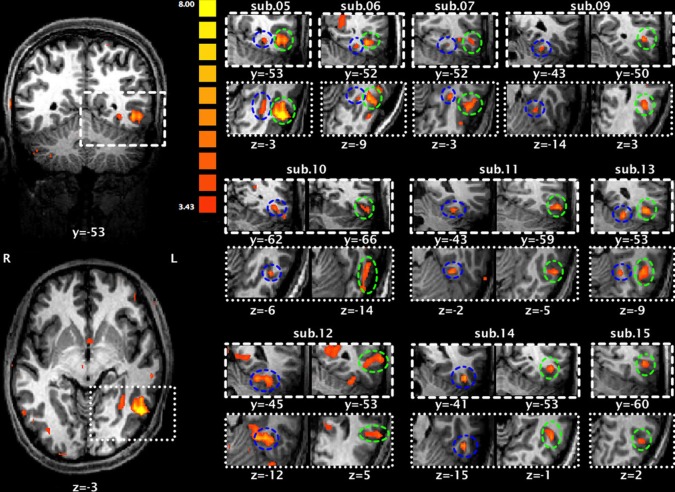
**Significant activation of the left occipitotemopal cortex for each subject during localizer runs for Chinese characters compared to faces**. The two activation maps on the left (upper: coronal view; lower: horizontal view) are from subject 5. Maps on the right are each subject's activation map superimposed on that individual's anatomical map. Blue circles indicate activation of the left occipitotemporal sulcus (LOTS) and green circles indicate activation of the left inferior temporal gyrus (LITG).

Figure 3 presents brain maps showing significant activations for Chinese characters compared to faces in localizer runs for each subject, and the selection of each individual's ROIs (also see Table 3 for their peak coordinates). These two regions were adjacent, with the loci for LOTS activation more mesial and inferior to those for LITG activation. This pattern was highly consistent across subjects.

**Table 3:** **Two regions of interest (ROIs) showing significant activation for Chinese characters compared to faces**.

**Subject no**.	**LOTS**			**LITG**		
	**Voxels**	***T*-value**	**Peak (*x, y, z*)**	**Voxels**	***T*-value**	**Peak (*x, y, z*)**
S5	163	5.07	−29, −52, −1	659	11.18	−48, −58, −2
S6	54	4.37	−27, −52, −8	438	6.80	−35, −52, −8
S7	280	5.08	−30, −40, −8	242	6.41	−36, −58, 4
S9	80	4.28	−28, −41, −12	129	5.34	−45, −48, 4
S10	152	5.32	−40, −62, −5	285	6.11	−49, −51, −11
S11	98	5.42	−32, −42, −11	163	5.86	−50, −57, −4
S12	683	6.74	−32, −45, −12	346	5.05	−55, −51, 8
S13	152	6.25	−23, −51, −9	397	6.51	−44, −53, −5
S14	157	6.45	−43, −40, −12	286	5.77	−44, −52, 0
S15	–	–	–	178	5.05	−46, −58, 5
Average	202	–	−32, −47, −9	312	–	−45, −54, −1

LOTS, left occipitotemporal sulcus; LITG, left inferior temporal gyrus; Talairach coordinates; T-test, all p < 0.01.

**Figure 4:**
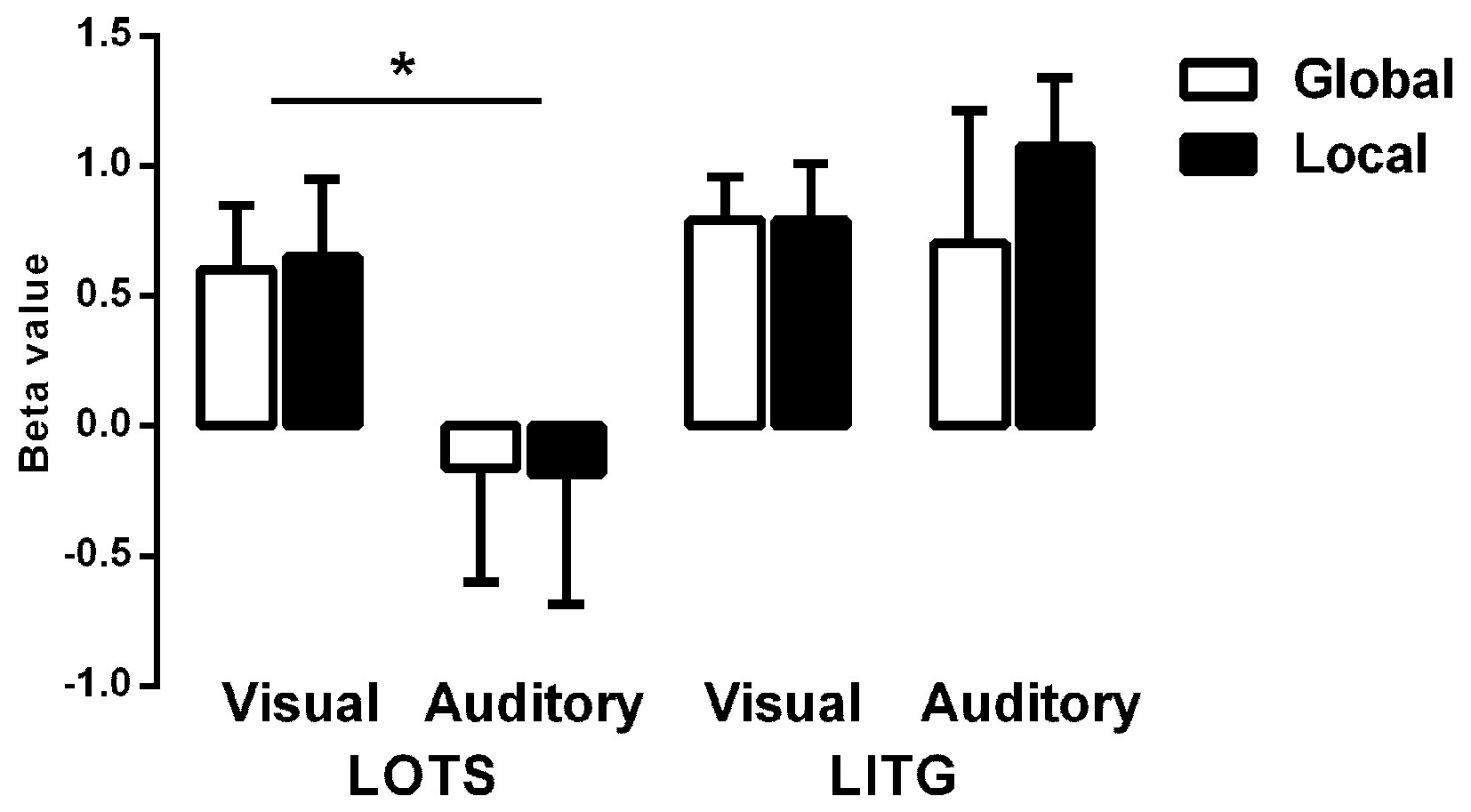
**Average Beta values for each ROI and for each condition**. LOTS, left occipitotemporal sulcus; LITG, left inferior temporal gyrus. ^*^p < 0.05.

Figure 4 shows the average beta values of both word-specific regions (ROIs) for each experimental task (Lv, Gv, La, Ga). A significant main effect of region [*F*_(1, 8)_ = 13.77, *p* < 0.01] and a region by modality interaction [*F*_(1, 8)_ = 64.23, *p* < 0.001] were found. *Post-hoc* analysis revealed that the LITG was significantly activated for both visual-based and auditory-based tasks, but that LOTS was significantly activated only by visual-based tasks [*F*_(1, 8)_ = 10.64, *p* < 0.05], indicating that LOTS may be a modality-specific region, while LITG may be a multimodal region. Surprisingly, there were no significant main effects or two/three way interactions for processing-level factors, suggesting that different levels of linguistic processing, either phonological or orthographic, did not significantly modulate activation level within either ROI.

## Discussion

The current study took advantage of the unique characteristics of the Chinese writing system to examine the functional properties of the VWFA. Current findings showed that there were two regions in the left ventral occipitotemporal cortex showing selective activation for Chinese characters. One region was the LOTS and the other was the LITG. However, they responded differently to inputs depending on modality. The LOTS responded exclusively to visual inputs, while the LITG showed comparable responses to both visual and auditory inputs. Accordingly, the LOTS may serve as a modality-specific region and can be regarded as the VWFA for Chinese reading, while the LITG may serve as a multimodal region analogous to the LIMA/BTLA.

This activation pattern for Chinese processing coincides with findings from previous studies on alphabetic languages (Jobard et al., 2003, 2007, Cohen et al., 2004). However, the loci of the modality-specific and multimodal regions were anatomically distinct from the locations reported in previous studies on alphabetic languages. In the current study, LOTS activation (Talairach Coordinate, TC -32, -47, -9) was slightly mesial and superior to the VWFA identified in previous studies on alphabetic languages (Cohen et al., 2000, 2002, TC -42, -57, -15; Jobard et al., 2007, TC -48, -56, -12). The coordinates of the multimodal region (LITG, TC -45, -54, -1) was slightly mesial and superior to the corresponding region found in previous studies on alphabetic languages (Cohen et al., 2004, TC -58, -56, -8; Jobard et al., 2007, TC -50, -44, -10).

Even among studies on Chinese language processing, the location of the VWFA has been inconsistent. Three recent studies employing the same localizer technique as used in the current study reported VWFA locations relatively close to that reported here (TC -38, -49, -12 in Ma et al., 2011; TC -45.4, -51.5, -9.1 in Bai et al., 2011; TC -43.8, -55.6, -8.8 in Xu et al., 2012). In contrast, a meta-analysis study concluded that the coordinates of the VWFA for Chinese characters deviated less than 5 mm in each dimension compared to that for English words, suggesting a consistent localization of VWFA across writing systems (Bolger et al., 2005). Moreover, other studies have also localized the VWFA for Chinese characters closer to that for alphabetic languages (Xue et al., 2006; Liu et al., 2008; Mei et al., 2010; Song et al., 2012). To our knowledge, there were no similar findings regarding to LITG multimodal region for processing of Chinese character have been reported in the literatures.

In summary, although current findings indicated that there is a functional VWFA and a lateral inferior temporal multimodal region for both alphabetic and logographic writing systems (functional reproducibility), these regions may occupy slight different regions of the cortex (i.e., no anatomical reproducibility). Deviation in VWFA locations could reflect differences in the visual features of different writing systems, the principles of form-to-sound mappings, top-down modulation, and (or) task requirements at the linguistic level. Therefore, future studies should directly compare processing of alphabetic and logographic characters in bilingual subjects to explore different organizational patterns in the left ventral temporal cortex and the underlying mechanisms (e.g., whether the VWFA loci differ due to different processing requirements).

In addition, Cohen et al. (2004) has proposed the anterior part of the superior temporal sulcus (STS) as a possible unimodal auditory area. In the current study, auditory-based tasks exclusively activated the left supermaginal gyrus and the right posterior STS, but there was no anterior STS activation in either hemisphere. However, visual and auditory tasks showed overlapping activation in the left angular gyrus, which includes the posterior part of the STS. This finding is in accord with that of Price et al. (2003). This area is generally considered as a multimodal region, responsible for integrating visual and auditory inputs (Price, 2000; Booth et al., 2002). Therefore, the proposal of “an auditory equivalent of the VWFA” by Cohen et al. (2004) requires further investigation.

Unexpectedly, the current study showed that different levels of phonological or orthographic retrieval did not influence the activation of word-specific regions (LOTS or LITG), suggesting that the VWFA may be involved in form-to-sound mapping at the syllable-level for Chinese reading. However, there are other possibilities. First, processing level may influence the inter-regional connections at the network level rather than at the individual regional level (Bitan et al., 2005; Deng et al., 2012). However, how task requirements modulate intra-regional activation is still unclear. A recent study demonstrated that task requirements modulated the activation intensity of the VWFA (Wang et al., 2011). In contrast, it has also been reported that the spatial profile of response selectivity in the left inferior temporal cortex is not modulated by attentional levels (Xu et al., 2012) or task requirements (Ma et al., 2011). Second, the difficulty of the current tasks may have influenced the result. On one hand, task difficulty varied across the four tasks as evidenced by differences in accuracy and RT (with the La task being the most difficult). However, it is uncertain if task difficulty affects local activation of language-related regions. In several studies, increased difficulty of a reading task did not increase activation of language-related areas but rather activated additional regions associated with attention, memory, and executive function (Gur et al., 1988; Paus et al., 1998; Drager et al., 2004). On the other hand, a difficult task *per se* may change the subjects' strategies for performing the task (Huber, 1985). Therefore, it is difficult to exclude the possibility that the current tasks, especially the auditory ones, may be completed without substantial orthographic processing. As a result, modulation of task requirements may have a major influence on additional aspects of cognitive processing (e.g., discrimination, working memory) dependent on other brain regions, rather than on orthographic analysis. Additional experiments are needed to explore how psycholinguistic variations, especially within the same domain (e.g., phonological retrieval), influence spatial representation and response specialization of the left occipitotemporal cortex for language processing.

### Conflict of interest statement

The authors declare that the research was conducted in the absence of any commercial or financial relationships that could be construed as a potential conflict of interest.
