# Supplementary material for: Unimodal and multimodal regions for logographic language processing in left ventral occipitotemporal cortex
Source: Front Hum Neurosci. 2013 Sep 27;7:619. doi: 10.3389/fnhum.2013.00619 (PMC3784977; doi:10.3389/fnhum.2013.00619)
Supplement: Supplementary file 1 [file DataSheet1.PDF]

Supplementary Table 1. The list of all stimuli for four tasks.

Lv			Gv			Ga & La			
Ch.	Pinyin	Freq.	Ch.	Pinyin	Freq.	Ch.	Pinyin	Freq.	*Homophone (Freq.)*
班	ban1	432.4	背	bei4	221.8	草	cao3	417.9	
般	ban1	550.2	泵	beng4	254.8	车	che1	763.0	*砗(0.1)*
残	can2	145.5	策	ce4	275.8	扯	che3	19.5	
缠	chan2	17.9	臭	chou4	30.8	抽	chou1	131.8	*瘳(0)*
淡	dan4	74.0	废	fei4	129.7	揣	chuai1	0.2	*啜(0.2)*
烦	fan2	28.5	粪	fen4	67.9	寸	cun4	268.2	
犯	fan4	94.0	奉	feng4	23.1	打	da3	765.6	
敢	gan3	151.2	惠	hui4	34.4	等	deng3	1,931.0	*戥(0.1)*
韩	han2	43.6	货	huo4	133.3	短	duan3	354.1	
喊	han3	89.1	禁	jin4	57.3	发	fa1	3,167.7	
坎	kan3	23.5	旧	jiu4	357.2	放	fang4	1,113.6	
满	man3	339.3	俊	jun4	18.7	肥	fei2	415.9	*腓(0.5)*
难	nan2	501.7	酷	ku4	30.6	佛	fo2	30.1	
盼	pan4	10.2	括	kuo4	165	改	gai3	854.6	
散	san4	266.9	类	lei4	744.4	告	gao4	217.6	*诰/锆(0.2/2.7)*
杉	shan1	17.5	掠	lue4	38.4	更	geng4	876.4	
陕	shan3	35.4	律	lv4	295.2	怪	guai4	61.6	
坛	tan2	15.0	梦	meng4	37.1	海	hai3	621.5	*胲(0)*
探	tan4	148.5	命	ming4	2615.4	吼	hou3	7.4	*醢(0)*
晚	wan3	267.4	牧	mu4	87.6	缓	huan3	99.0	
办	ban4	574.8	逆	ni4	67	卷	juan3	296.5	*锩(0)*
蚕	can2	64.3	弄	nong4	124.4	卡	ka3	197.2	*咔/佧/胩(0/0/0)*
产	chan3	4319.4	确	que4	648	口	kou3	684.6	
丹	dan1	83.7	热	re4	870.7	苦	ku3	403.9	
番	fan1	39.5	润	run4	241.3	捆	kun3	12.6	*悃/阃(0)*
函	han2	50.7	弱	ruo4	195.1	浪	lang4	82.5	
寒	han2	130.1	释	shi4	110.4	冷	leng3	265.4	*阆/蒗/莨(0/0/0.2)*
看	kan4	1291.8	竖	shu4	16.8	俩	lia3	31.9	
览	lan3	52.6	顺	shun4	168.8	拎	lin1	1.7	
蛮	man2	17.3	素	su4	423.1	卵	luan3	217.3	
南	nan2	787.2	特	te4	1072	乱	luan4	126.3	
攀	pan1	19.0	替	ti4	154.1	论	lun4	1,757.6	
然	ran2	1342.5	戏	xi4	80.1	买	mai3	97.2	
扇	shan4	28.5	幸	xing4	40.5	面	mian4	2,801.4	*眄(0)*
善	shan4	147.8	续	xu4	506.1	命	ming4	2,615.4	
贪	tan1	37.1	训	xun4	285	摸	mo1	73.3	
弯	wan1	106.8	意	yi4	1271.3	某	mou3	299.7	
完	wan2	792.2	硬	ying4	225.4	拿	na2	192.9	*镎(0)*
赞	zan4	127.6	狱	yu4	10.6	闹	nao4	77.5	*淖(0.3)*
展	zhan3	1338.0	助	zhu4	376.3	弄	nong4	124.4	
沉	chen2	136.1	池	chi2	140.3	暖	nuan3	53.2	
独	du2	236.9	楚	chu3	161.4	女	nv3	266.9	*钕(0)*
规	gui1	579.0	登	deng1	100.4	胖	pang4	9.4	

挥	hui1	240.9	夺	duo2	226.2	跑	pao3	143.8	
敬	jing4	34.1	耕	geng1	144.1	拼	pin1	23.5	*姘(0)*
孔	kong3	1181.4	鼓	gu3	304.5	品	pin3	671.9	*榀(0.1)*
扩	kuo4	192.8	壶	hu2	12.1	巧	qiao3	41.6	*愀(0)*
配	pei4	533.7	棵	ke1	27.3	切	qie1	943.6	
歧	qi2	30.8	脊	ji3	63.9	且	qie3	614.5	
射	she4	362.0	绩	ji1	106.4	圈	quan1	194.9	*悛(0.2)*
舒	shu1	44.4	皆	jie1	37.4	犬	quan3	8.4	*畎/绻(0/0)*
活	huo2	1080.7	肯	ken3	69.2	让	rang4	294.9	
刑	xing2	21.0	孙	sun1	85.2	惹	re3	5.6	
谊	yi4	58.1	哭	ku1	18.1	扔	reng1	16.0	
幼	you4	204.6	虏	lu3	10.2	仍	reng2	201.0	
约	yue1	391.4	旅	lv3	52.9	日	ri4	1,499.9	
辈	bei4	39.7	捏	nie1	14.7	肉	rou4	86.5	
笔	bi3	83.1	妻	qi1	12.1	散	san4	266.9	
等	deng3	1931.0	秦	qin2	103	傻	sha3	9.5	
盾	dun4	267.5	辱	ru3	15.8	晒	shai4	36.3	
革	ge2	2552.5	施	shi1	518	少	shao3	1,000.8	
骨	gu1	294.1	鼠	shu3	132.5	舍	she3	50.8	
季	ji4	285.0	提	ti2	1289.3	收	shou1	513.6	
聚	ju4	120.6	亭	ting2	11.6	爽	shuang3	8.6	
决	jue2	1073.3	投	tou2	242	说	shuo1	2,720.1	
蒙	meng2	75.1	涂	tu2	93	团	tuan2	959.1	*抟(0)*
普	pu3	368.1	妥	tuo3	39.2	腿	tui3	53.3	
禽	qin2	11.0	维	wei2	486.6	娃	wa2	14.3	
森	sen1	65.6	温	wen1	736.7	歪	wai1	47.2	
耸	song3	12.0	喜	xi3	130	外	wai4	1,301.0	
委	wei3	866.6	歇	xie1	25.7	我	wo3	4,114.0	
屋	wu1	86.1	协	xie2	221.7	用	yong4	4,730.2	
雪	xue3	98.1	熊	xiong2	23.3	晕	yun1	10.2	*氲(0)*
英	ying1	403.0	许	xu3	477.7	咱	zan2	172.7	
誉	yu4	15.1	疑	yi2	78.6	怎	zen3	377.6	
尊	zun1	91.6	隐	yin3	39.2	窄	zhai3	35.0	
损	sun3	220.9	庸	yong1	38.8	抓	zhua1	371.5	
雄	xiong2	245.1	珍	zhen1	37.8	准	zhun3	492.7	
铁	tie3	566.5	执	zhi2	230.3	总	zong3	919.4	*偬(0)*
陡	dou3	18.4	总	zong3	919.4	走	zou3	557.0	
Mean Freq.:		*371.5*			*249.8*			*578.9*	*0.1*

Ch.=characters; Pinyin=Chinese pronunciations (numbers indicate tone); Freq.= written frequency (per million characters). Homophones are for those stimuli in Ga and La task only.
